# Mr. Ring, on the Kensington Case

**Published:** 1804-12-01

**Authors:** John Ring

**Affiliations:** New Street, Hanover Square


### *To the Editors of the Medical and Physical Journal.*

GENTLEMEN,

IN the last Number of the Medical and Chirurgical Review, the account of the Kensington case is considerably corrected. It is there acknowledged, that Mr. Cullurne never saw the patient after the fourth day; and I have the authority of that gentleman for asserting, that he did not form any decisive opinion on the case; on the contrary, he desired the child might be brought to him again for this purpose.

The author of the account in the Med. and Chir. Review confesses, that Mrs. Meredith prevaricated in her evidence; and endeavours to reconcile it to reason by saying, that she was *minutely* and *unexpectedly* questioned on points of some nicety, with which she must be supposed not to have been familiar. The public, however, will judge whether this is not the best mode of eliciting the truth; and it must be recollected, that during the first examination, she positively asserted the colour was not black, and persevered in that assertion.

It was an easy matter for her afterwards to learn what the colour of the cow-pock scab is, either from conversation with her numerous visitors, some of whom were not very friendly to vaccination; or from some vaccine patients in the neighbourhood, whom she repeatedly saw about that period.

In the account alluded to, the most material circumstance is artfully suppressed; namely, that the pustules were rubbed off within a few days after their appearance. "On the above evidence," it seems, lame and imperfect as it is, "the public may judge for themselves," whether the patient had the cow-pock. It is easy to conjecture, what opinion the author of the article alluded to wishes the public to form; but they will be cautious how they believe one, who has already tried to deceive them. *Stulto non creditur etiam verum dicenti.*

A person, *assuming the style of an Editor*, in the same number of the Medical and Chirurgical Review, affects to *fear*, what he evidently *hopes*, that the cases which have lately occurred, will induce many families to prefer the inoculation of the small-pox to vaccination. This person, who is probably the same that drew up the false account of the Kensington case, appears to be envious of the well-earned fame of Dr. Jenner; and anxious to pluck the laurels from his brow. He would sacrifice vaccination to accomplish his purpose. He thinks vaccine inoculation cannot be established by Dr. Jenner, and a few of his adherents. He thinks Dr. Jenner's adherents are *interested*. They would, however, have given greater reason for that reproach, if they had been so mean as to sell cow-pock matter.

This Writer, and a Reviewer in the same work, have drawn a conclusion rather unfavourable to vaccination from Dr. Rollo's report of experiments at Woolwich. From the same premises, a Reviewer in the Medical and Physical Journal has drawn a favourable conclusion. Having been requested by Dr. Rollo to observe the progress of those cases, I deem it incumbent on me to point out an inaccuracy in his statement.

When I received from Dr. Rollo a letter mentioning the alarm created in his mind by the effects of variolous inoculation on those whom he had before vaccinated, I wrote to him a letter expressive of my opinion, that his alarm would prove groundless. Mr. Knight, to whom Dr. Rollo also applied, wrote to him an answer to the same purport.

When

When I waited on Dr. Rollo, I remarked to him and several other gentlemen present, that the local pustule of the patients under the test of variolous inoculation was different from that which takes place in persons who have not had the cow-pock or the small-pox; and that it was not surrounded by a cluster of pustules, as it is in those who have not undergone either of those disorders.

This remark was not controverted at the time; I could not, therefore, but be rather surprised at seeing it stated in Dr. Rollo's Report, that in one of his cases a cluster of pustules appeared. It is true, the local affection was rather more than common; probably owing to the heat of the weather, and to the parts being much handled.

In order to ascertain with as much precision as possible the particulars of these cases, I wrote to Mr. Butler, surgeon, of Woolwich, whose children were the subjects of two of them; and received an answer to the following effect:

"The arms of these children were frequently examined by medical men, which must have produced some irritation; the children themselves more particularly irritated them by scratching."

Mr. Butler says, there were several pustules near the inoculated part, in Serjeant Gloag's child; and that, when examined with a glass, they appeared to contain *a watery fluid.*

This proves that they were vesicles, the mere effect of cuticular inflammation; a pustule contains pus. It is much to be wished, that medical men would be a little more accurate in their discriminations.*

Mr. Butler observes, that he feels it difficult to describe the few pimples, or pustules, which appeared on his own child. "When first noticed they felt hard; and, on the third or fourth day, when examined with a glass, they appeared to contain a small quantity of a watery fluid. None of them were ever so large as those which occur in the small-pox."

Notwithstanding Mr. Butler speaks with so much modesty, I defy the most able nosologist to give a better idea of a miliary eruption. Sauvage says, it is "an eruption of blisters not larger than a millet seed." Linnæus calls it "a round papulous eruption." Vogel describes it to be "pustules of the size of millet seeds, full of serum, which is at first limpid, afterwards whitish, and of a pearl colour." Sagar represents it to be "an eruption of blisters seldom larger than a millet seed." He says, "they remain on the skin four, five, six, seven, nine, eleven days, or more, and terminate in fine branny scales." Cullen tells us, it is "a small papulous eruption; and that, in a day or two, a very small white pustule, of short duration, is visible on its apex."

Nothing can bear a more exact resemblance to the eruptions which are continually mistaken for the small-pox; particularly by those who make experiments with variolous matter. In confirmation of this opinion it must be recollected, that matter taken from such eruptions never produces any specific disease. Ex nihilo nihil fit.

Dr. Rollo and Mr. Butler both informed me, that in the cases where variolous matter was inserted as a test, the pustule appeared earlier than usual. The same, it is well known, commonly happens in those who have previously had the small-pox; but to this rule there are many exceptions.

Dr. Rollo, in his letter to me, stated, that he thought his patients going on in the manner described by Baron Dimsdale in his first twelve cases. This was to me a strong argument that they were insusceptible of the general small-pox, and that the variolous inoculation would prove abortive; as it appears to have done in almost every one of the twelve cases related by Baron Dimsdale. That great inoculator has described the effects of variolation in those who have already had the disease; but he did not know how to account for them. This was reserved for Dr. Woodville, and other later authors.

It is now well understood, that the early efflorescence, and the premature pustule, such as are to be met with in the works of Baron Dimsdale, Mr. Goldson, Dr. Rollo, and others, are nothing more than the mountain in labour; and they would probably terminate in the same manner, did not the troublesome itching attendant on such cases provoke the patients to scratch their arms, and curiosity tempt practitioners to press them; hence a train of local and general symptoms in proportion to the injury received.

In Dr. Rollo's two first cases, there was an efflorescence round the puncture, about the size of a sixpence, on the second day. In the third case, the elevation and inflammation of the arm were still more remarkable than in the two former. On the eighth day the inflammation nearly extended to the elbow, and upper part of the arm; and over the whole side. It is no wonder the child was *feverish*; and, although it had no eruption, it may be said to have have escaped as through fire. So far is the variolous test from being a matter of indifference, as some people pretend.

In the fourth case, the child was inoculated with variolous matter in the evening; and by the next morning, his arm was inflamed nearly to the size of a sixpence. He complained of its itching much; and said it was very sore. In the evening the inflammation was greatly increased. On the third day it was still farther increased; and had considerable hardness round it. On the fourth day the extent of the inflammation was three inches by two. The child complained of pain under his arm, and in the evening was much indisposed. On the morning of the fifth day the inflammation was abated, and the child much better. This was one of Mr. Butler's children, part of whose case has already been related. Several of the vaccine patients whom Dr. Rollo inoculated with variolous matter, were affected with general indisposition; reasons for which may easily be found in the violence of the local inflammation.

In the fifth case there was a considerable inflammation on the arm by the next morning, and it increased till the fifth day. On the ninth day it again increased considerably, attended with all the symptoms of fever, and a convulsion. Some small eruptions appeared near the inoculated part; but none that could be called pustules; otherwise matter would have been taken from this child, as it was from his brother, although it proved ineffectual for the purpose of inoculation.

These are sufficient specimens of Dr. Rollo's experiments with variolous matter after vaccination. Any one who examines the result of his experiments, in children who had not previously undergone vaccination, will perceive a material difference in them. In these cases there was nothing doubtful, nothing equivocal; but an inflammation more tardy, and pustulous eruptions that bore every stamp of the small-pox.

Upon the whole, Dr. Rollo is inclined to think, that the cow-pock is endowed with a power of resisting the small-pox; but Mr. Butler is a more decided advocate for the practice. He assured me, when I was at Woolwich, that nothing had so strongly convinced him of the efficacy of vaccination, as the result of the preceding experiments. In his letter to me he declares, that he has a perfect confidence in vaccination; and thinks it his duty to recommend the practice to the public.

Dr. Rollo having excited a local variolous pustule in Mr. Butler's children, inoculated other children with some of the matter thus produced, and excited the small-pox in its usual form. This, as he confessed to me, was one circumstance which staggered him. In his report he says, these children were inoculated from pustules on the arms of those who had undergone vaccination; but in the analysis of his publication, in the Med. and Chir. Review, it is justly remarked, that it should have been stated, whether the matter was taken from the inoculated part, or from one of the secondary pustules. I therefore think it necessary to mention, that it was taken from the inoculated part. "It has been ascertained," as the Reviewer truly asserts, "that a pustule may be excited in a person who has gone through the small-pox; and that the matter of such pustule is capable of giving infection.

Of this I informed Dr. Rollo; and, at the same time, expressed my regret, that he had propagated the infection thus generated. It was this circumstance that spread an alarm far and wide; and gave rise to the remark I had made in my answer to Mr. Goldson, previous to the publication of the Review in question, and even of Dr. Rollo's Report. It is as follows:

"The possibility of having a local variolous pustule after the small-pox, or the cow-pox, having been often ascertained, I hope that in future, when gentlemen meet with such cases, they will not deem it necesary, or even justifiable, to put the matter to the test, by inoculating with that deadly poison any persons who have not yet had the disease.

"Such an experiment, when matter is taken from a pustule in one who has been previously vaccinated, not only tends to spread the contagion of the small-pox, but also to excite a doubt of the efficacy of vaccination in the mind of the public."

Dr. Rollo took matter for inoculation from two secondary eruptions on Master Butler, but it proved unsuccessful. Had any other eruptions appeared more promising, there is no reason to doubt that he would have taken matter from them also.

The writer in the Med. and Chir. Review, speaking of the cases in Fullwood's Rents, says, "These cases have unfortunately followed close on the heels of the unfavourable ones of Dr. Rollo, a man of acknowledged ability as an observer." Those who acknowledged the ability of Dr. Rollo as an observer, must allow that his evidence is in favour of vaccination; since he was not able to observe a single variolous eruption, in any one of those vaccine patients whom he inoculated for the small-pox.

The Reviewer of Mr. Dunning's pamphlet, in the same number of the Review, has provided an antidote for the poison, which this malignant writer is endeavouring to instil into the public mind. He very judiciously remarks, that in the aforesaid "pamphlet, several cases are given at length, where the patients were subjected to variolous inoculation after having been vaccinated from three to five years before. *The effects corresponded very nearly with those observed by Dr. Rollo to have occurred in his experiments; that is, a good deal of local inflammation was excited in the inoculated parts; and in some an eruption of pimples took place, preceded by constitutional affection. The precise characters of small-pox were, however, wanting.*"

This proves, that the statement of the other writer in the same Review is a misrepresentation; which, "*following close on the heels*" of the misrepresentation of the Kensington case, indicates a deliberate design to calumniate vaccination. Whether the author of those misrepresentations is an original self-interested opponent of the practice, or a jealous rival of Dr. Jenner, I shall not pretend to determine; but I trust, that one channel at least will always be open, in which the calumnies of prostitute prints may be refuted; otherwise we may see another string of falsehoods, such as lately appeared in the Times, where it was asserted that a child was vaccinated two years before she was born.

It cannot have escaped notice, that in the narrative alluded to, the names of some respectable practitioners were joined with others of a very different description, in order to give currency to the statement; but this was done without their consent.

---

I now beg leave to offer a few observations on Dr. Macdonald's Answer to Mr. Goldson, published in your Journal for October; which I consider as a valuable acquisition to Medical Science. In some respects, however, the opinions of Dr. Macdonald are different from those which are commonly entertained.

He thinks it still doubtful, whether it is requisite that the matter of grease should pass through the nipple of the cow, in order to produce the desired effect in the human body. This point, I apprehend, numerous experiments, particularly those of Drs. Loy, Sacco, La Font, and De Carro, have satisfactorily decided in the negative.

Dr. Macdonald is of opinion, that the matter of grease, if inserted into the nipple of a mare, would produce nothing. He says, the experiments instituted by Professor Vibourg have clearly proved that the grease is not infectious in the horse. A negative, however, is not so easily proved; otherwise we could demonstrate, that the cow-pock does not originate from grease. The difficulty lies in procuring the genuine matter of grease in an active state. This matter succeeds, when inserted into the nipple of a cow; and the late Mr. Davy shewed Dr. Jenner and me an instance, in which vaccine matter succeeded when inserted into the heel of a horse. I therefore see no reason to doubt, that active equine matter would succeed, when inserted into the nipple of a mare.

Dr. Macdonald says, medical records abound with cases of spurious eruptions, produced by the variolous contagion. Among these spurious eruptions he reckons the chicken-pox. To such an hypothesis, I conceive, the members of the medical profession in general will not assent. I have alluded to it in my Treatise on the Cow-pox, p. 943 and 975; but little expected, that it would have met with so respectable an advocate as Dr. Macdonald.

Dr. Macdonald contends, that several cases of eruption subsequent to vaccination, found in Dr. Woodville's Report, have erroneously been taken for the small-pox from previous infection; of which he thinks the efflorescence a sufficient proof. I have, however, seen exceptions to this rule. Dr. Macdonald thinks the eruptions which happened subsequent to vaccination, in the practice of Dr. Ballhorn and Mr. Stromeyer at Hanover, were the fruits of exposure to variolous infection, since the small-pox was in the same neighbourhood, and even in the same house; but he considers the disease thus produced, to have been the chicken-pox.

That the pustulous disease produced in the vaccine patients in the Small-pox Hospital was the small-pox, I can safely aver; matter derived from that source having excited the small-pox in my own practice, and in that of many others; but, with regard to the Hanoverian cases, I must confess, upon minutely reconsidering them, some appear to have been variolous, and some varicellous. In the former there was a hard base, and a suppuration at the apex; in the latter a vesicle.

That the former was really the small-pox could not be proved, unless matter had been taken and used for inoculation; lation; but every thing which I have observed relative to the chicken-pox convinces me, that the hypothesis of its originating from degenerate small-pox matter is ill founded. This idea probably took its rise from the very frequent mistake of the chicken-pox for the small-pox.—Hence virus taken from the former, and inserted instead of the latter, producing its like, induced practitioners to suppose, *or to pretend,* that the small-pox sometimes degenerates into the chicken-pox.

Were this the case, the chicken-pox, as an epidemic, would more commonly follow than precede the small-pox; a circumstance which I have never been able to ascertain; and I am fully persuaded, that one of those diseases as often happens first, as the other.

---

Having lately seen a coexistence of the chicken-pox and the scarlatina, I embrace the present opportunity of recording the fact. It occurred in the family of Mr. Child, in Barlow's Buildings, Long Lane, Southwark. This case afforded an additional proof of the compatibility of two morbid actions; both diseases having appeared at the same time, and pursued their natural course. The scarlatina was extremely violent; but terminated well.

---

Some gentlemen, rather sanguine in their hopes, and ostentatious in their reports, wish the world to suppose that they have extended the practice of vaccination to New South Wales; letters lately received from Mr. Savage, late assistant surgeon of the Glatton, now settled at Parramatta, prove that this desirable object is not yet accomplished.

From one of his letters, received by Mr. Harwood, formerly surgeon of the Providence, it appears, that on his leaving England, he intended to have preserved his vaccine matter by inoculating passengers on board the ship; but, owing to a mistake or misrepresentation, this privilege was afterwards refused.

This letter was dated Rio Janeiro, December 8, 1802. He there tells us, that the medical practitioners, and inhabitants in general, wished much to have vaccination introduced among them; but he was afraid to open the packet of matter he had received from me, as they were to remain main on that station only a week, lest it should be injured by the admission of air. This I mention, for the sake of informing any persons who may in future touch at any distant port, that a week, and even a much shorter time, is sufficient to excite a fresh supply of matter; which will not only enable mariners to leave this boon behind them, but also facilitate its conveyance to the place of their destination.

By a letter which I received from Mr. Savage, dated Sydney, New South Wales, May 13, 1803, I was informed that he had commenced his experiments with vaccine virus; but from subsequent intelligence it appears, they proved unsuccessful. It has been commonly supposed, the small-pox has never visited that remote region; but Mr. Savage declares there are sufficient traces of it among the natives, to warrant him in believing it has existed there. Should it ever appear in that part of the world again, he has no doubt but, from the heat of the climate, and the intemperance of the inhabitants, its ravages will be dreadful; unless prevented by the grand prophylactic which has immortalized a Jenner.

The last letter received from Mr. Savage, dated Parramatta, February 29, 1804, states, that Dr. Anderson was about to send a surgeon from Madras, with vaccine virus, and some children; in order to introduce vaccination into New South Wales. This plan holds forth the most flattering prospect of success.

Mr. Savage notices one circumstance relative to the climate of New South Wales, which is equally singular and surprising. The changes from heat to cold, and from cold to heat, are sudden and frequent; insomuch, that it is not uncommon, during the land-wind, for the thermometer to stand at upwards of 100° in the shade; and by a sudden shift of wind to the southward, for it to fall instantly to 50° or even below it. Yet these changes are not found to be productive of any injurious consequences. The climate is healthy; and the soil fruitful.

I am, &c.

JOHN RING.

*New Street, Hanover Square.*

